# Hybrid 3D ranging and velocity tracking system combining multi-view cameras and simple LiDAR

**DOI:** 10.1038/s41598-019-41598-z

**Published:** 2019-03-27

**Authors:** Neal Radwell, Adam Selyem, Lena Mertens, Matthew P. Edgar, Miles J. Padgett

**Affiliations:** SUPA, School of Physics and Astronomy, University of Glasgow, Glasgow, G12 8QQ UK

## Abstract

Scanning our surroundings has become one of the key challenges in automation. Effective and efficient position, distance and velocity sensing is key to accurate decision making in automated applications from robotics to driverless cars. Light detection and ranging (LiDAR) has become a key tool in these 3D sensing applications, where the time-of-flight (TOF) of photons is used to recover distance information. These systems typically rely on scanning of a laser spot to recover position information. Here we demonstrate a hybrid LiDAR approach which combines a multi-view camera system for position and distance information, and a simple (scanless) LiDAR system for velocity tracking and depth accuracy. We show that we are able to combine data from the two component systems to provide a compound image of a scene with position, depth and velocity data at more than 1 frame per second with depth accuracy of 2.5 cm or better. This hybrid approach avoids the bulk and expense of scanning systems while adding velocity information. We hope that this approach will offer a simpler, more robust alternative to 3D scanning systems for autonomous vehicles.

## Introduction

As technology advances, more and more emphasis is being placed on automation. Sensors are crucial to give automated systems enough information to make good decisions. For an automotive vehicle the essential information is a 3D map of its surroundings and therefore developing techniques to map spatial position as well as depth has become a key challenge^1–3^. The challenge is to develop a 3D imaging system cost-effective enough to place on a car, with a range of up to 100 m, depth accuracy resolution in the millimeter to centimeter range and sufficient transverse spatial resolution to classify objects. Existing ranging technologies such as radar can easily achieve the range but the long wavelength limits the transverse spatial resolution of scenes^4^, while ultra-sonic imaging technology has the transverse resolution but lacks range^5–7^.

Light detection and ranging (LiDAR) has become the leading technology in this application area. LiDAR operates on the time-of-flight (TOF) principle, where short pulses of light illuminate the scene and a fast detector measures the return delay to sub nanosecond precision^8–10^. The delays reveal the depth information as longer delays correspond to objects which are further away. Building up a full 3D map of the scene requires an imaging modality which can attribute this depth information to a specific point in the scene. The simplest approaches use scanning spots^11,12^, which build up an image of the scene one voxel at a time, which can be further improved by scanning several spots at once.

Though technically simple, scanning techniques have limitations^13^. Scanning is typically achieved with moving mirrors, increasing the size and cost of systems while also limiting acquisition time. Laser power is also limited by eye-safe levels of exposure, and concentrating power into a spot minimises the total usable laser power. Spot scanning is also incompatible with compressed sensing techniques, as one always needs to scan every point to build an image. Several of these limitations can be overcome using structured illumination LiDAR^14–17^. However, current implementations of those systems remain bulky and costly. A simpler approach to 3D scene reconstruction is stereo or multi-view camera systems^18^ which use multiple camera images to triangulate points in a scene, and though advanced calibration methods exist^19^ these methods struggle with absolute depth registration while also being computationally expensive.

Here we demonstrate a hybrid approach to 3D scene reconstruction where we combine a simple scanless LiDAR system with multi-view camera technology. The core principle is to remove the complexity, bulk and cost of providing spatial resolution from a LiDAR system by having that task performed by a multi-view camera system. A LiDAR system which simply flood illuminates the scene provides the absolute depth accuracy which is lacking from the camera system and additionally provides velocity information. The disparate information from both systems is then compared and fused to provide one final composite image with spatial(x and y), depth(z) and velocity information. This system is completely passive, removes the need for scanning lasers and can be achieved with cheap camera technology, resulting in a system which is robust, cost-effective and compact. We hope that this technology can help reduce the cost and complexity of collision avoidance systems in automated vehicles.

## Experimental Setup and Methods

The experimental setup comprises a multi-view camera rig and a LiDAR system with associated timing electronics. The LiDAR system provides a depth histogram for the entire scene while the mutli-view cameras provide an uncalibrated 3D point cloud. After data acquisition the data fusion step reconciles both data streams into a single composite image combining position, depth and velocity information into a single output image.

### LiDAR System

**Figure 1:**
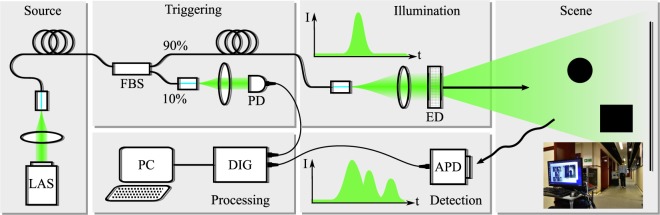
Experimental Setup. LAS: 532 nm pulsed laser, FBS: 90:10 Fibre beamsplitter, PD: Trigger photodiode, ED: Engineered diffuser, APD: Avalanche photodiode, DIG: Digitiser, PC: Computer. Lower right inset photo shows a static corridor scene.

The LiDAR system is shown in detail in Fig. 1 and consists of several sub-systems. The light source consists of a pulsed laser coupled into a multimode fibre beamsplitter. The laser is a 532 nm solid state passively Q-switched laser (Teem Photonics SNG-03E-100) with 700 ps pulses at 7 kHz rep. rate and 3 *μJ* pulse energy. The triggering system is used to prime the timing electronics to measure pulse delays. The 90:10 beamsplitter (TM105R2F1A) sends 10% of the light to a photodiode (PDA100A-EC) which is connected to the trigger input of the digitiser (NI PXIe-1071 with NI PXI-5154). The other 90% of the light passes to the illumination section where it is collimated (F220FC-532) and diffused by an engineered diffuser (ED1-S50) with diffusion angle chosen to match the field of view (FOV) of the multi-view camera system. The laser pulses illuminate and scatter from the scene and are detected by a silicon avalanche photodiode (APD, Menlo Systems APD210) in the detection section. The APD has a 1 GHz 3 dB bandwidth (1.6 GHz max bandwidth) and 0.5 mm active area diameter. Even though this is a relatively large area detector, using a lens to match the large FOV of the illumination and camera systems is difficult, requiring a very short focal length lens and precision alignment. We therefore do not use a lens and simply collect light as it falls on the detector. The signals are digitised by a digital oscilloscope (PXI 5154) with 2 Gs/s sampling rate. Sampling multiple pulses and using random interleaved sampling mode gives an effective sampling rate of 20 Gs/s (50 ps time bins). Each light pulse triggers an acquisition via the trigger photodiode, with wires and electronics introducing some unknown delay. This is calibrated out by first converting the LiDAR temporal signals to depth signals through the speed of light. A screen is then placed exactly 1 m away from the system and the signal delay is tuned until the peak falls at exactly 1 m.

Each LiDAR distance signal is then averaged over 5 acquisitions to reduce noise. Velocity measurements require subsequent measurements of the depth signal which we take by recording a group of 5 LiDAR signals at a fixed time delay of 100 ms. This group of 5 signals then undergo data fusion processing described later.

### Multi-view Camera System

**Figure 2:**
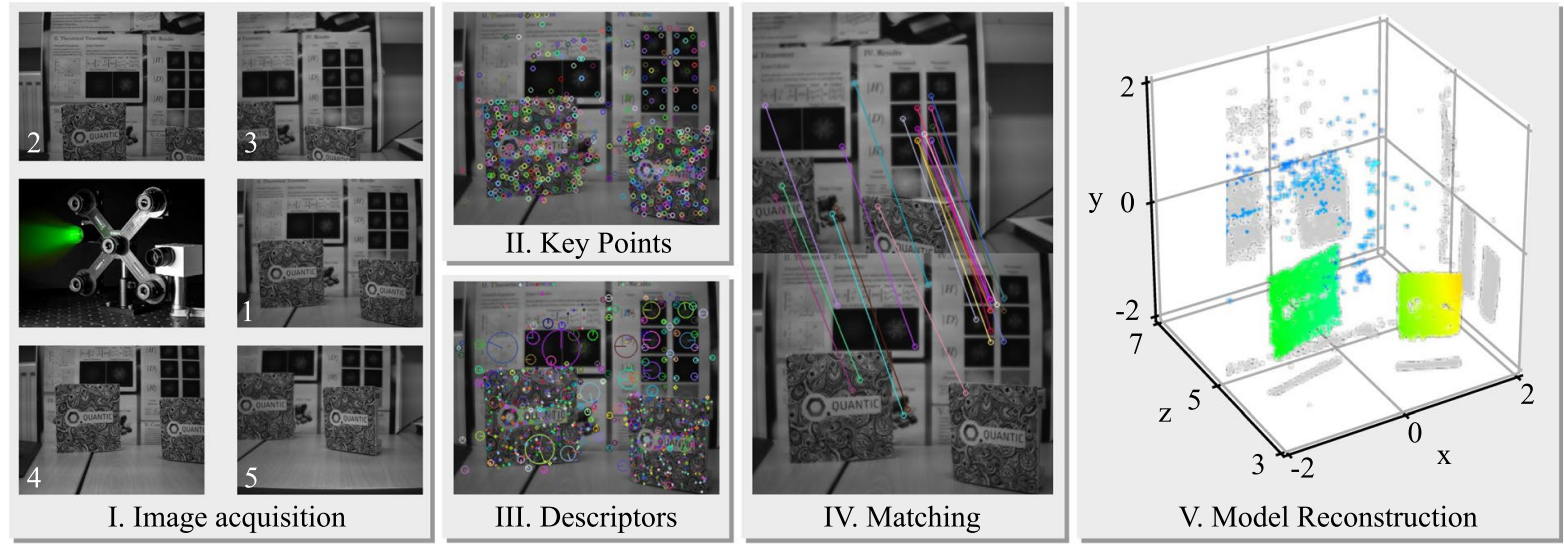
Multi-view camera process. Stage I: Images are simultaneously acquired from 5 cameras using the setup shown in middle left image. Stage II: Keypoints are identified. Stage III: Scale and rotationally invariant key point descriptors are calculated. Stage IV: Each image pair has its features compared to find matches. V. Bundle adjustment compares the matches and finds camera positions and a 3D model which minimises the model errors.

The multi-view camera system is an array of 5 USB cameras (Thorlabs DCC1545M) producing 1280 × 1024 resolution images using 12 mm focal length lenses (MVL12WA) giving a FOV of 38.5° (see Fig. 2 inset). The star pattern (with opposite corner camera separation of 20 cm) is chosen to have a central camera as the master image for the final composite image. 5 cameras are chosen to improve the quality of the 3D reconstruction while maintaining acceptable reconstruction times and the camera separation is chosen to provide suitable range while remaining compact. Images are acquired by a high-performance desktop computer (6-core Intel i7-8700K with 4000 MHz RAM and a Tesla K40c graphical compute unit) for reconstruction and data fusion.

Several methods are available for reconstructing 3D scenes from multiple images. Methods which mimic human sight look for the relative shift of an object from 1 camera (eye) to the other. The shift, or ‘disparity’, is typically measured by matching sub-sections or ‘blocks’ of the images along the epipolar line and directly reveals depth information. Disparity measures such as the semi-global block matching algorithm^20,21^ are simple yet computationally intensive and can be difficult to make robust to a variety of scenes.

More advanced 3D scene recovery methods instead characterise the ‘features’ of the images and an example reconstruction pipeline is illustrated in Fig. 2. Images are acquired and key points (edges and corners) are identified. Feature descriptors are then calculated by characterising the area around each key point. Advanced feature descriptors such as those from the scale invariant feature transform (SIFT) algorithm are scale and rotationally invariant, allowing feature matching between images taken from different distances and perspectives.

The feature descriptors are then compared or ‘matched’ between the images, linking the features in each image and their 3D positions are then triangulated. When more than two images are used, each pair of images will provide a 3D model, which will disagree. One solution is to allow the model to adjust the assumed camera positions and properties while minimising the error in the models. This ‘bundle adjustment’ approach has the major benefit of not requiring any calibration, as the camera parameters are not input into the system but rather come out of it.

Here we use Changchang Wu’s ‘VisualSFM’^22–24^ software which implements SIFT feature matching and a parallel bundle adjustment algorithm for model reconstruction, both of which are optimised to run on a graphics processing unit (GPU). Images are acquired simultaneously in LabVIEW and VisualSFM can perform the entire computation pipeline, from images to a 3D model, or we can interject with our own data or reconstruction algorithms. The final output is a 3D point cloud of each feature in the scene.

### Data Fusion

The LiDAR system provides temporal signals with no spatial information while the multi-view camera system provides an uncalibrated 3D point cloud. The data fusion step combines these data sources into a single composite image of the scene with accurate spatial, depth and velocity information.

**Figure 3:**
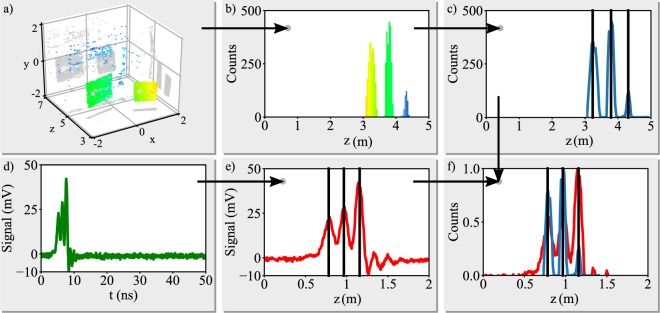
Data fusion process. Top row: The 3D point cloud (**a**) is converted to a depth histogram (**b**) and peaks are identified (**c**). Bottom Row: The LiDAR time signal (**d**) is converted to depth and peaks are identified (**e**). A polynomial function maps the two sets of peaks (**f**) and scales the point cloud data.

The first step is to spatially scale the uncalibrated 3D point cloud data to match the LiDAR signal, and is outlined in Fig. 3. The point cloud data (Fig. 3a) is first converted to a depth histogram (Fig. 3b), where peaks are detected (Fig. 3c). The temporal LiDAR signal (Fig. 3d)) is then converted to depth data via the speed of light and a number of peaks are detected to match the previously fitted point cloud data (Fig. 3e), with this constraint chosen as the depth resolution of the 3D point cloud data does not suffer from the peak broadening arising from the non-zero pulse width as is the case with the LiDAR signal. The positions of both sets of peaks are used to calculate a scaling function (a polynomial of order *N* − 1 where *N* is the number of peaks) which overlaps the peaks (Fig. 3f). The example in Fig. 3 was fitted with the function

1 $$y={a}_{1}{x}^{2}+{a}_{2}x+{a}_{3}$$

where *a*_1_, *a*_2_ and *a*_3_ are the fit parameters and *x* and *y* are set to the 3 peak positions for the point cloud and LiDAR peak positions respectively. The 3D point cloud x, y and z coordinates are then scaled by this scaling function.

The second stage then makes a new histogram of the scaled 3D point cloud depths where the bin number and widths are chosen to exactly match the sampling rate of the LiDAR signal and the peak finding, matching and scaling is repeated for a better match. The 3D point cloud data then has its x and y coordinates zeroed by subtracting the mean x and y values.

**Figure 4:**
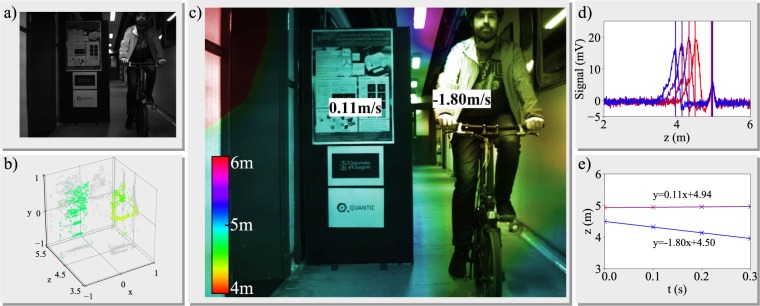
Composite LiDAR depth map. (**a**) Central camera image. (**b**) 3D Point Cloud. (**c**) Composite image, depth is mapped to colour using a hue colourmap. Velocity labels are placed at the central location of each depth peak. (**d**) Consecutive LiDAR signals, vertical lines placed at the positions where peaks are found. (**e**) Position of the peaks found in (**d**) with linear fit to infer velocity, annotated with best fit equation.

The last stage of the data fusion is to produce a composite image, illustrated in (Fig. 4). The central camera image (Fig. 4a) is used as the master image, and we set the hue value of each pixel to represent the depth information from the scaled point cloud (Fig. 4b), where the period of the hue can be set based on the application. We know the pixel location of each point in the point cloud, however this is still a rather sparse representation of the depth, as many pixels have no corresponding feature and therefore no depth information. To assign each pixel a depth, we use nearest point interpolation, chosen to minimise calculation time. The hard boundaries arising from this interpolation are then softened using a Gaussian smoothing pass.

Velocity information is then extracted by tracking peak positions from subsequent LiDAR acquisitions with a fixed time interval, here chosen to be 100 ms (Fig. 4d). A linear fitting of these peak positions reveals their velocities (Fig. 4e), which we then super-impose onto the composite image using a label (Fig. 4c). To position the label we must infer the transverse position of each peak. A depth threshold is set, and each point in the 3D point cloud is assigned to a peak if its z coordinate is within the depth threshold of the peak depth. The central coordinate of each object is then calculated and the velocity label is placed there, completing the composite image.

### Depth Accuracy

Assessing the accuracy of the depth information requires comparison with some ‘ground-truth’ data. For our analysis we choose to analyse the flatness of the poster board within the data. Under the assumption that the poster board is in reality a flat surface, then the depth accuracy can be estimated by the deviations of the recovered 3D data for the poster board from a plane. The points which should represent the poster board are isolated from the other data using a simple depth threshold. These points are then fitted to a plane of the form *z* = *Ax* + *By* + *C*, with the constants *A*, *B* and *C* recovered using a least squares method. The distance d_i_ of the ith point **P**_**i**_ = (**p**_**xi**_, **p**_**yi**_, **p**_**zi**_) from the plane is calculated by

2 $${d}_{i}=({{\bf{P}}}_{{\bf{i}}}-{{\bf{P}}}_{{\bf{0}}})\cdot \hat{{\bf{m}}},$$

where **P**_0_ = (0, 0, *C*) is an arbitrary position on the plane and $\hat{{\bf{m}}}=(A,B,-1)/\sqrt{{A}^{2}+{B}^{2}+1}$ is the unit vector of the plane. The vector (**P**_i_ − **P**_0_) joins a point to the plane and *d* is obtained by taking its projection along the plane normal. The ‘depth accuracy’ *δ* for *N* points is then defined to be

3 $$\delta =\sqrt{\frac{\sum _{i=1}^{N}{d}_{i}^{2}}{N-1}},$$

which is the standard deviation of the distances of the points from the plane.

## Results

**Figure 5:**
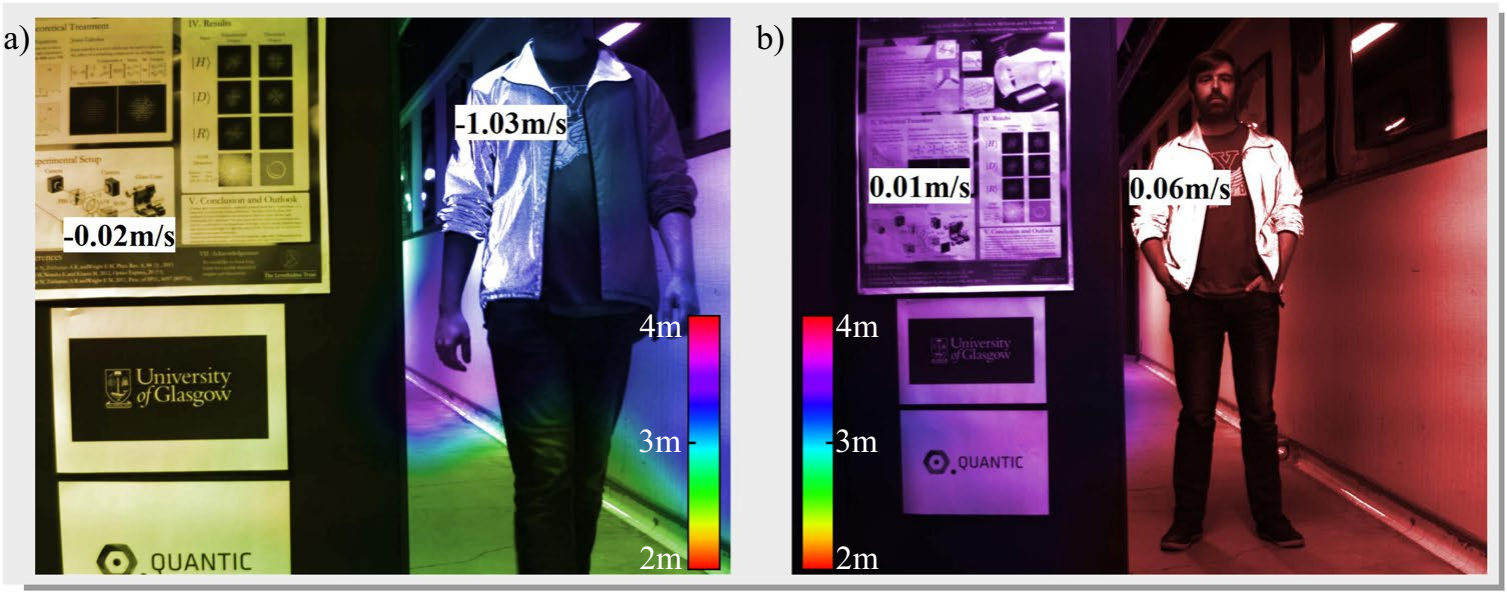
Composite images for walking and stationary scenes. Both images are treated as in Fig. 4(c).

Our proof-of-principle Hybrid LiDAR system has been tested on three corridor scenes, a stationary target, a walking target and a cycling target, all taken relative to a stationary poster board for depth accuracy estimation. Figure 4c shows the composite image for the cycling scene and the point cloud in Fig. 4b clearly shows a good representation of the cyclists arms, head and torso as well as the bike handle bars. The poster board is also well represented and the colours in the composite image reflect this. The depth accuracy, detailed in subsection 1.4 is 1.8 cm at a distance of 4.97 m (0.4%). The results in Fig. 4e show an excellent velocity fit, however the error on the poster board velocity is 0.11 m/s, which we attribute to peak position drift due to noise. The results for walking and stationary target are shown in Fig. 5 and show an improved velocity accuracy for the stationary targets. The depth accuracy is 2.5 cm at 2.39 m (1.0%) and 2.2 cm at 3.59 m (0.6%) for the walking and stationary results respectively. The inverse relationship between distance and depth accuracy indicates that the underlying depth error is absolute, rather than relative and is less than 2.5 cm.

## Discussion

The system runs continuously with a new composite image produced at 1–2 Hz. The primary limitation of acquisition rate is the multi-view 3D reconstruction time. In particular, the SIFT feature finding algorithm and bundle adjustment model reconstruction are computationally expensive, with a single reconstruction taking of order 10 s on a standard laptop. One of the main motivations to use VisualSFM is that it integrates both the SIFT and bundle adjustment algorithms on GPU, and by harnessing the computational power of a 6-core Intel i7 8700 K coupled to a Nvidia Tesla K40c GPU we are able to reduce this reconstruction time to below 1 s. This hardware is somewhat at the limits of what is currently available, but with the advancement of GPU processing, new feature finding approaches and the ever decreasing cost of computation power, reconstruction times could approach video rates in years to come.

Reconstruction time can also be minimised by down-sampling the camera images, adjusting the SIFT parameters, or using a faster feature finding algorithm such as ORB^25^. All of these methods however result in fewer features and therefore 3D points, reducing the fidelity of the result. This is a trade-off which is particularly undesirable as the system is already susceptible to poor fidelity when the scene is relatively featureless. As an example, the point cloud in Fig. 4b shows a lot of data around the poster and cyclist, but provides no information at all about the wall or floor, as these surfaces lack features around which to triangulate. However, for natural, busy scenes this problem will be mitigated. Additionally, this system still performs well in situations in which a conventional scanning LiDAR system might struggle, in particular scenes filled with specular reflections such as a scene with many cars, pose problems for conventional LiDAR systems as the high signals from reflections blind the system. Our system is more robust to these scenes as the camera systems can still function while locally saturated. In this sense, this approach can add robustness through diversity where multiple approaches can be used to cover the weaknesses of each other.

We have shown results in the 3 to 5 metres range, where the system performs well. Results are still possible at 8 m but the signal-to-noise ratio of the detected signals become marginal. Laser power and detector size and sensitivity are the key limits. Lasers in the near infrared allow higher laser powers while remaining eye-safe, however this requires a move away from silicon detectors resulting in poorer sensitivity and reduced active area. Newly emerging technology in time-correlated single photon counting (TCSPC) electronics retain or improve on the temporal resolution while also allowing detection of a single scattered photon, offering a route to increasing the range of these technologies towards 100 m.

The camera separation or baseline should also be increased to increase the range. The cameras are more-or-less scale invariant and we suggest that a 20-fold increase in baseline from 10 cm to 2 m (the width of a car for instance) would increase the effective range to at least 100 m.

## Conclusion

We have demonstrated a hybrid LiDAR system combining multi-view 3D imaging with a simple LiDAR system, which produces composite images in which position, depth and velocity data are presented. A 5 camera multi-view system recovers a 3D point cloud of the scene using GPU accelerated feature finding, matching and model reconstruction. This point cloud data is fused with depth information from simple scanless LiDAR system which also provides a measure of velocity. We have demonstrated 3D imaging performance for indoor corridor scenes, for which we were able to reconstruct 3D objects around feature rich areas of the scene with depth accuracy of 2.5 cm. The system produces composite images at a rate of 1–2 Hz at a range of 5 m, limited by computational and laser power respectively. This completely passive system could find applications in collision avoidance in automotive and aeronautical applications.
